# Flavin Mononucleotide-Based Fluorescent Proteins Function in Mammalian Cells without Oxygen Requirement

**DOI:** 10.1371/journal.pone.0043921

**Published:** 2012-09-11

**Authors:** Janine Walter, Sascha Hausmann, Thomas Drepper, Michael Puls, Thorsten Eggert, Marcel Dihné

**Affiliations:** 1 Department of Neurology and Epileptology, Hertie Institute for Clinical Brain Research, Eberhard-Karls-University, Tübingen, Baden-Württemberg, Germany; 2 evocatal GmbH, Düsseldorf, North Rhine-Westphalia, Germany; 3 Department of Neurology, Heinrich-Heine-University, Düsseldorf, North Rhine-Westphalia, Germany; 4 Institute of Molecular Enzyme Technology, Heinrich-Heine-University, Düsseldorf, Forschungszentrum Jülich, Jülich, North Rhine-Westphalia, Germany; Center for Genomic Regulation, Spain

## Abstract

Usage of the enhanced green fluorescent protein (eGFP) in living mammalian cells is limited to aerobic conditions due to requirement of oxygen during chromophore formation. Since many diseases or disease models are associated with acute or chronic hypoxia, eGFP-labeling of structures of interest in experimental studies might be unreliable leading to biased results. Thus, a chromophore yielding a stable fluorescence under hypoxic conditions is desirable. The fluorescence of flavin mononucleotide (FMN)-based fluorescent proteins (FbFPs) does not require molecular oxygen. Recently, the advantages of FbFPs for several bacterial strains and yeasts were described, specifically, their usage as a real time fluorescence marker in bacterial expression studies and their ability of chromophore formation under anaerobic conditions. Our objective was to verify if FbFPs also function in mammalian cells in order to potentially broaden the repertoire of chromophores with ones that can reliably be used in mammalian studies under hypoxic conditions. In the present study, we demonstrate for the first time, that FbFPs can be expressed in different mammalian cells, among them murine neural stem cells during proliferative and differentiated stages. Fluorescence intensities were comparable to eGFP. In contrast to eGFP, the FbFP fluorescence did not decrease when cells were exposed to defined hypoxic conditions neither in proliferating nor in differentiated cells. Thus, FbFPs can be regarded as an alternative to eGFP in studies that target cellular structures which are exposed to hypoxic conditions.

## Introduction

Since its first description and purification, the green fluorescent protein (GFP) from *Aequorea victoria* [1] and its mutant forms [2], [3] became widely used tools in molecular biology for various applications. The enhanced green fluorescent protein (eGFP) has been used for studying gene expression, drug discovery or developmental mechanisms as well as graft behaviour [4], [5], [6]. However, the expression of the eGFP gene and fluorescence development is strictly depending on molecular oxygen as the α,β bond of tyrosine 66 needs to be oxidized during the self-catalyzing process of chromophore formation [7]. As a consequence, hypoxic conditions notoriously reduce eGFP fluorescence, substantially limiting its application. For this reason it was also noted that eGFP might be unsuitable for the expression in obligate anaerobic bacteria and yeasts [7]. To overcome this limitation, a novel class of FMN-based fluorescent proteins (FbFPs) was developed by genetically engineering the bacterial blue-light sensitive light oxygen voltage domains (LOV) of the photoreceptors YtvA from photoreceptors YtvA from *Bacillus subtilis* [8] and SB2 from *Pseudomonas putida* [9]. It could be shown, that in contrast to the members of the GFP family, FbFPs are suitable fluorescent reporter proteins for quantitative analysis of microbial processes in the presence or absence of oxygen [10], [11], [12], [13], [14]. To this point, the usage of FbFPs is not fully characterised in eukaryotic cells. Specifically, FbFP functioning in mammalian cells is unknown, although important applications under oxygen limiting conditions are obvious: Tissue and cell oxygenation is of great interest in several fields of biomedical research like organ transplantation [15], host and parasite interaction [16] stroke research [17], [18], tinnitus [19], ischemic eye disease [20], myocardial ischemia [21] and, in particular, oncology [22], [23], [24]. Since *in vivo* or *in vitro* models of these diseases or conditions are connected to primary hypoxia or secondary ischemic hypoxia, reporter proteins should also reliably function during hypoxic conditions. For instance, several studies report hypoxia-induced changes in proliferation and differentiation of tissue specific stem cells and human embryonic stem cells directly illustrating the necessity for stable reporter proteins also in stem cell research if those populations are supposed to be investigated under hypoxic conditions [25], [26], [27], [28]. It has been reported that there is a significant loss in eGFP fluorescence of up to 40% in mammalian cells if cultured for 12 hours at an oxygen saturation below 0.02% [29]. And it is known that different tumor cell lines tolerate an oxygen saturation of 0.01–0.3% for 12–24 hours [29], [30], [31]. Thus the intensity of the eGFP fluorescence might decrease under these culture conditions. In contrast to tumor cells, it is known that 30–50% of cells within populations of different neuronal cell lines die when exposed to these hypoxic conditions for 12 hours [32], [33]. Notably, oxygen saturations of 0.3, 0.7 or 1.4% were detected *in vivo* in viable human tumors [34], [35], [36]. These values correspond to an oxygen partial pressure of 2.5, 5, or 10 mmHg (millimeter of mercury), respectively, at 37°C. In comparison to these hypoxic values, physiological mammalian arterial blood oxygen partial pressure lies between 80 to 100 mmHg and the oxygen partial pressure under standard cell culture conditions is up to 142.6 mmHg [37] illustrating that mammalian tumors can tolerate considerably inhospitable environments and thus need robust reporter proteins.

Cellular structures of interest, although potentially surviving a situation of critical oxygen supply, would stand to lose their accessibility to analyses when the intensity of the eGFP signal decreases under hypoxic conditions. We therefore investigated if FbFPs can be used in mammalian cells as suitable reporter proteins under oxygen limitation.

## Results

### Comparative expression of FbFPs and eGFP in different mammalian cells

**Figure 1:**
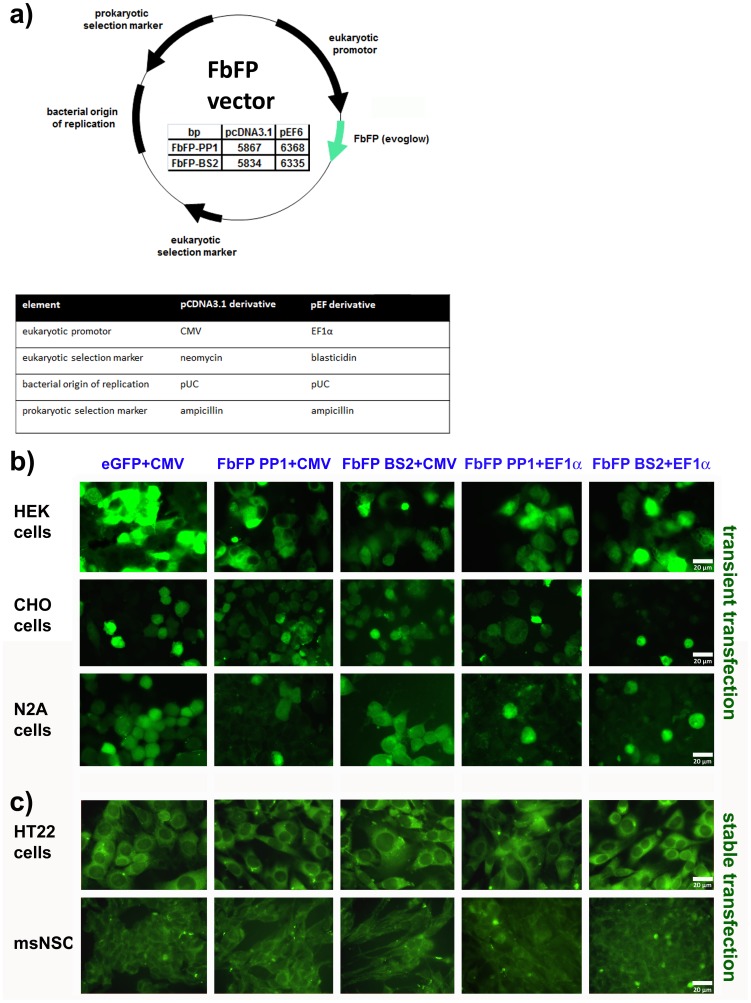
Transient and stable transgene expression of the flavin mononucleotide-based fluorescent protein FbFP in tumor cells and mouse neural stem cells. In a: schematic drawing of the FbFP vector is shown. The table in the vector map summarises the amount of base pairs and the plasmid backbones (pcDNA3.1 and pEF6) of al vectors used in this study (PP1 and BS2). In b: transiently transfected tumor cells lines from different species are shown (HEK cells; human embryonic kidney cells, CHO; chinese hamster ovarial cells, N2A; mouse neuroblastom-2a). The fluorescence of two different FbFPs (PP1 and BS2) each with the CMV or the EF1α promoter is compared to the fluorescence of eGFP (with CMV promoter as control). In c: two stably transfected neural cell populations are shown. In the upper row, a tumor cell line (HT22) and, in the lower row, mouse ES-cell derived neural stem cells (msNSC) are given. The fluorescence of two different FbFPs (PP1 and BS2) each with the CMV or EF1α promoter is compared to the fluorescence of eGFP (with CMV promoter as control).

To evaluate, if PpFbFP (PP1) and EcFbFP (BS2) can be used as fluorescent reporter proteins in mammalian cells, we first generated recombinant derivatives of plasmid vectors pcDNA3.1 or pEF6 carrying the respective reporter genes (Fig. 1, a). For efficient expression, the codon usage of FbFP genes was optimized and fused to the Kozak consensus sequence. The resulting expression vectors were subsequently used to investigate if mammalian cells are principally able to express FbFPs. Therefore, we transiently transfected HEK, CHO or N2A cells with plasmids encoding two different FbFPs (PP1 or BS2), respectively. Each plasmid contained the corresponding FbFP gene under the control of CMV or EF1α promoter (Fig. 1, a). To control the efficiency of the transfection and to compare FbFP and GFP fluorescence intensities, we used a standard eGFP vector with CMV promoter mediating eGFP expression. Our results demonstrated, that (i) transfection of eGFP- or FbFP-encoding vectors occurred with comparable efficiencies and (ii) the expression strength (fluorescence intensity) as well as cytosolic localization of FbFP proteins in tumour cell lines determined by visual inspection is similar compared to eGFP after transient transfection (Fig. 1, b). In order to investigate the fluorescence stability in cell populations that consistently express the transgene, we performed a selection of those cells that were stably expressing the transgenes by antibiotic treatment. Therefore, cells were kept under selective growth conditions with the respective antibiotic. Fluorescence brightness was first measured 24 and 48 hours after transfection, subsequently the fluorescence was verified by visual inspection every week. By this procedure we were able to generate cell populations that stably express FbFP under the CMV or EF1α promoter or eGFP under the CMV promoter over 10 to 20 passages. We could show that the fluorescence intensity determined by visual inspection within tumor cell lines or murine neural stem cells stably transfected with FbFP is similar compared to that of cells stably transfected with eGFP (Fig. 1, c). To further investigate if FbFP applicability varies in dependency on the maturation level of the cell population and to monitor expression levels in a broad range of different cell types, we also performed transient transfections of the FbFP variants PP1 and BS2 in murine embryonic fibroblast cells (MEF cells), murine ES cells (msESC) and HeLa cells. We found that the FbFP-mediated fluorescence intensity was similar compared to that of eGFP in all cell types studied (data not shown).

### Effects of oxygen limitation on FbFP and GFP fluorescence

**Figure 2:**
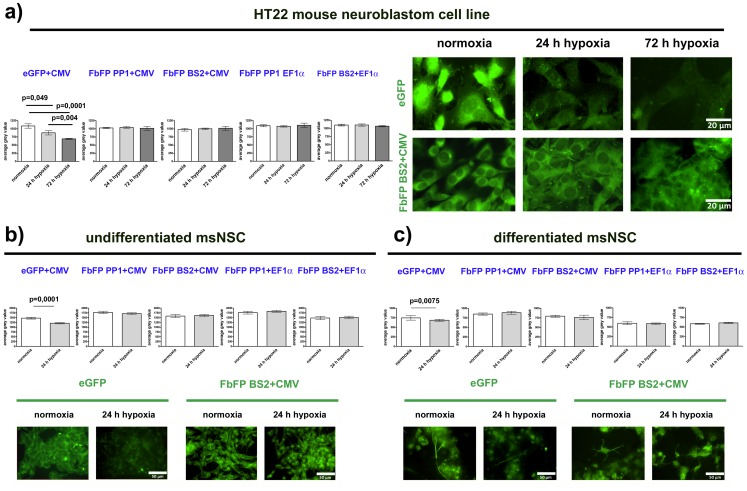
Fluorescence intensity of FbFPs under normoxic and hypoxic conditions. In a: The left panel shows average grey values as an indicator for fluorescence intensity in the HT22 cell line under normoxic conditions and after 24 or 72 hours of hypoxia. The right panel shows photomicrographs of stably transfected HT22 cells at indicated oxygen saturations. In b+c: average grey values in undifferentiated msNSCs or differentiated msNSC under normoxic conditions and after 24 or 72 hours of hypoxia are shown together with photomicrographs of stably transfected cells at indicated oxygen saturations. P-values are given for results of unpaired two-tailed t-test results of normoxic vs. hypoxic conditions. White bars, light or dark grey bars illustrate values under normoxic conditions, 24 or 72 hours hypoxia, respectively.

To verify if the intensity of FbFP fluorescence is independent from molecular oxygen, we quantified the FbFP (PP1, BS2) and, for control, eGFP fluorescence under normoxic and hypoxic conditions. For this purpose, first, the average grey values as indicators for fluorescence intensities of HT22 cells, undifferentiated or differentiated msNSCs (each stably tranfected with eGFP or FbFP PP1 or BS2 under CMV or EF1α promoter) under normoxic conditions were determined. Afterwards, the average grey values of each group were determined after 24 or 72 hours under hypoxic conditions (Fig. 2, a–c).

The presented values under normoxic conditions are relative average grey values, different intensities in the three examined cell types arise from their different protein expression levels. Notably, the baseline fluorescence intensities of eGFP and FbFPs under normoxic conditions nearly reached the same relative levels in stably transfected cell lines, whereas the baseline fluorescence intensity of FbFPs in transiently transfected cell lines was lower in comparison to eGFP.

After tracing the baseline fluorescence intensities, we exposed all cell types to hypoxia for 24 or 72 hours and again recorded fluorescence intensities (Fig. 2,a). As expected, we were able to detect a significant decrease of nearly 20% in eGFP fluorescence in HT22 cells after 24 hours of hypoxia that became even more evident after 72 hours of hypoxia (reduction of ±40%). In contrast, the fluorescence intensity observed in HT22 cell lines that were stably transfected with FbFPs remained constant even after 72 hours of hypoxia. We confirmed the vitality of HT22 cells after hypoxia by visual inspection of cell morphology and DAPI nuclei staining to ensure that only fluorescence values of living cells were used for statistical calculations.

Murine ES cell-derived neural populations showed a much more sensitive reaction to hypoxia than tumor cells. After more than 24 hours of hypoxia, murine ES cell-derived neural populations began to die off. This phenomenon was observed by visual inspection and DAPI nuclei staining. Therefore, we were only able to study fluorescence stability of differentiated and undifferentiated msNSCs during a time interval of 24 hours of hypoxia. Like in the tumor cell lines, we were able to detect a significant decrease in eGFP fluorescence intensities after 24 hours of hypoxia in the undifferentiated neural stem cell stage as well as in mature cell populations whereas the fluorescence intensities of FbFPs remained unchanged (Fig. 2 b,c).

### Neuronal FbFP expression and its influence on the population size

**Figure 3:**
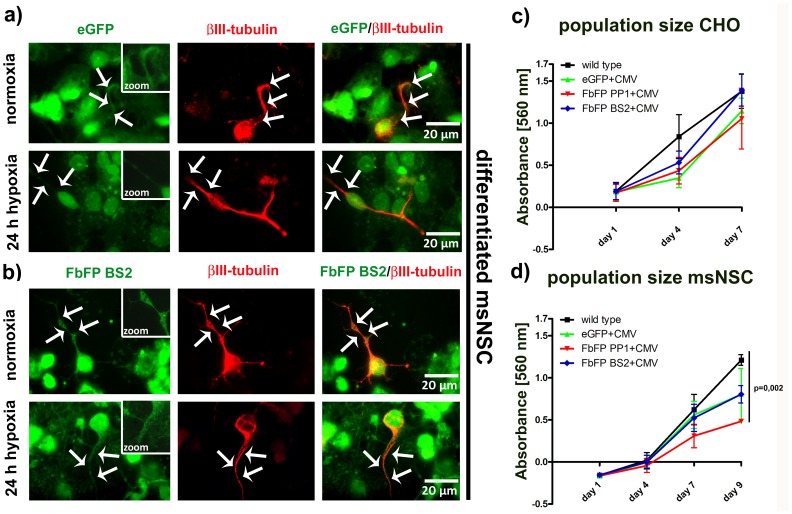
Neuronal transgene expression under hypoxic conditions and population size after stable eGFP or FbFP transfection. In a+b: To specifically verify neuronal transgene expression after neural stem cell differentiation, βIII-tubulin immuno-staining was applied. Photomicrographs in the first column show the transgene (green) eGFP or FbFP BS2. In the second column, βIII-tubulin immuno-staining (red) is shown and in the third column the merged picture. The transgene expression is illustrated under normoxic or hypoxic conditions. To better visualize transgene expression in critical structures in which the eGFP signal decreases under hypoxic conditions, white arrow heads are inserted that flank neurites of the neurons. Boxes show parts of neurites marked by the white arrow heads in a higher magnification. In c+d: Population size of stably transfected CHO cells and mouse neural stem cells are given. Cells were seeded at an equal density and monitored over a time period of 7 to 9 days (x-axis). Measurement values of the MTT-assay (y-axis) are given for each measurement day. Wild type cells were compared to stably transgene expressing cells.

To investigate the homogeneity of the FbFP signal within different parts of individual cells, we verified morphological aspects of neurons, whose small and ramified neurites are sometimes poorly labelled by the eGFP signal. For that, we performed immunocytochemical stainings against βIII-tubulin, a pan-neuronal marker, on stably transfected, differentiated msNSCs. We found that both types of fluorescent proteins showed a uniform distribution within the cytoplasm and neurites under normoxic conditions (Fig. 3, a and b). However, while the eGFP signal clearly decreases predominantly within neuritis under hypoxic conditions, the FbFP signal appeared to be stable here (Fig. 3, a and b, white arrowheads).

Then, to verify possible negative effects of FbFPs on the survival or proliferation of cells, we measured the size of the populations that were either stably transfected with one of the two different FbFPs (PP1 or BS2) under the CMV promoter in comparison to wild type cells or stably eGFP transfected cells under the CMV promoter. For this purpose, CHO cells or undifferentiated msNSCs were plated at the same densities and MTT-assays were performed 1 day, 4, 7 or 9 days after plating. CHO cells were only examined until day 7 due to their excessive proliferation. Notably, 9 days after seeding, FbFP PP1 transfected msNSC cells showed a reduced population size in comparison to wild type as well as FbFP BS2 or eGFP transfected cells. In CHO cells this phenomenon was not detectable (Fig. 3, d).

## Discussion

The light oxygen voltage domains (LOV) were described as blue-light sensitive photoreceptor domains in plants and prokaryotes. All LOV proteins bind FMN as chromophore and exhibit very low autofluorescence when excited by blue light at 450 nm [38], [39]. In plants they are associated to phototropism, the function in prokaryotes is associated to light reaction/sensing, this complex processes are still under scientific investigation [8], [40], [41], [42]. The LOV domains derived from *B. subtilis* and *P. putida* were genetically modified to increase the fluorescence in comparison to the wild type photoreceptors [12], [14]. Recently, these novel fluorescent reporters were used as molecular biology tools for hypoxic and anoxic investigations in several prokaryotic and eukaryotic organisms. They were used for host-microbe interaction studies [43] in *Porphyromonas gingivalis* and as gene expression reporters in *Bacteroides fragilis*, *Roseobacter* clade bacteria, *Candida albicans*, *Saccharomyces cerevisiae* and *E. coli* [10], [11], [13], [14], [44].

We were able for the first time to show, that oxygen independent FbFPs are suitable fluorescent reporter proteins in mammalian cells as well. After genetic modification of plasmid vectors and the usage of strong eukaryotic promoters, comparative expression studies revealed that the resulting visible fluorescence intensity of PP1 and BS2 after transfection and expression into mammalian cells was comparable to that of the widely used eGFP, although another study by Drepper *et al.* [12] found, that the total quantum yields of purified FbFPs were weaker than those of most GFP derivatives. Further it was possible to generate cell lines stably expressing FbFPs by antibiotic selection, which is important for long term experiments. The applicability of FbFP expression in mammalian cells was demonstrated for a variety of different tumor or immature cell lines, since we could show that next to CHO, HEK, N2A or HeLa cells, also pluripotent murine ES cells and murine ES cell-derived neural stem cells were transfectable with FbFPs. We could also show that neural stem cells maintained their FbFP fluorescence after differentiation and maturation and that they exhibited a uniform distribution of the FbFP signal in different cellular compartments, for instance, neurites. FbFP BS2 expression did not lead to alterations of the population size when compared to wild type or eGFP control. However, FbFP PP1 led to reduced population sizes in undifferentiated murine neural stem cells but not in CHO cells illustrating that FbFP PP1 might negatively influence the survival or proliferation of certain cell populations in contrast to FbFP BS2. This observation needs further exploration.

Most notably, we found that FbFPs are the more suitable fluorescence reporter under defined oxygen deprived conditions, in comparison to eGFP in mammalian cells. We were able to show that the fluorescence intensity of FbFPs remained stable under hypoxic conditions in comparison to that of eGFP which showed a reduction of up to 40%. We also investigated the fluorescence within neurites under hypoxic conditions in eGFP- or FbFP – transfected neurons and found that FbFPs more clearly label these structures. This directly illustrates that FbFPs can help to analyse such structures under hypoxic conditions. But also other applications of FbFPs in biomedical research during situations of critical oxygen supply are imaginable. In particular, cells that indeed suffer from low oxygen conditions finally being able to recover are now detectable during that critical period. For instance, in cancer research, transgenic labelling of the inner parts of solid tumors that are indeed at risk for necrotic transformation but still vital yet might be difficult as oxygen supply here is critical [45].

In the past, FbFPs were successfully used to visualize the anaerobic pathogens *B. fragilis* and *P. gingivalis* localized within human murine macrophages J774.1 and primary gingival epithelial cells, respectively without influencing the life cycle and the proliferative activity of these organisms [11], [43]. Thus, FbFPs could also be superior to label bacterial colonisations in the center of solid tumors, as bacteria were reported to be used as genetic vectors in anti cancer therapy [46], [47], [48].

However, as FbFP PP1 transfected msNSC showed a reduced population size, anti-proliferative and/or toxic properties of FbFP PP1 might be a shortcoming if mammalian neural stem cells are targeted by this fluorescent protein.

Another possible application is tracing the integration of experimentally transplanted cells into animal models for human diseases. For instance, stem cell-derived progeny is actually under investigation in the field of reconstructive neurobiology. As neovascularisation often lags behind proper graft integration, improved visualization of transplanted cells under situations of critical oxygen supply might be of substantial importance. And this might be true not only for studies in the field of stroke research.

It is further thinkable to use FbFPs as fluorescence reporter proteins in the species *Spinoloricus* nov. sp., *Rugiloricus* nov. sp., and *Pliciloricus* nov. sp., that were recently described to spend their entire live cycle under permanently anoxic conditions in the deep hypersaline anoxic basins of the Mediterranean Sea [49].

Taken together, our findings might pave the way for a completely new field of FbFP application in mammalian and other eukaryotic cells, specifically in biomedical research. We believe that after possible enhancements like improvement of photobleaching and quantum yields, as well as the generation of colour variants of FbFPs could enrich the field of molecular cell biology.

## Methods

### Cell culture, transfection and stable cell line generation

Tumor cell lines like human embryonic kidney cells (HEK cells), chinese hamster ovarial cells (CHO cells) or neuro 2a cells (N2A cells) and the murine, immortalized, hippocampal tumor cells line (HT22) were cultured in D-MEM High Glucose media (Invitrogen, Darmstadt, Germany) with 10% fetal calf sera (FCS) (PAA, Pasching, Austria). Mouse embryonic stem cell-derived (ES-derived) undifferentiated neural stem cells (msNSC) where cultured in D-MEM/F12 supplemented with N2-supplement (Invitrogen, Darmstadt, Germany), bovine serum albumin (BSA) (0.25% Equitech Bio, Kerrville, USA) and 20 ng epidermal growth factor and basic fibroblast growth factor (EGF and FGF) (Peprotech, Hamburg, Germany), respectively. Cell culture media contained Glutamax 20 mM (Invitrogen, Darmstadt, Germany), Penicillin 100 U/ml and Streptomycin 100 µg/ml (Invitrogen, Darmstadt, Germany). For differentiation of msNSC, the growth factors were withdrawn from the cell culture media. Cultivation of mouse embryonic stem cells and the generation of undifferentiated neural stem cells are described elsewhere [50], [51]. For transient and stable transfections of tumor cell lines, Attractene reagent (Qiagen, Hilden, Germany) was used. For stable transfections of neural stem cells, XFect Stem (Clontech/Takara Bio, Shiga, Japan) was used. For generation of stable cell lines (from cells transfected with a plasmid that contains the neomycin resistance gene), between 500 and 1000 µg/ml Geneticin was applied to the tumor cell culture. In neural stem cell cultures, the dosage of Geneticin was between 1 µg/ml and 2 µg/ml. For generation of stable cell lines (from cells transfected with a plasmid that contains the blasticidin resistance gene), 20 µg/ml Blasticidin was applied to the tumor cell culture. In neural stem cell cultures, the dosage of Blasticidin was 10 µg/ml. The cell lines used in this study were obtained from the American type tissue collection (ATCC, Manassas, USA).

### Induction of *in vitro* hypoxia

Hypoxia was generated by using a hypoxia incubator chamber (Billups-Rothenburg, Del Mar, USA) regarding to previous studies [31] and to the manufacturers instructions. Briefly, cells were placed in the chamber and a flow through of N_2_ gas was applied. The flow rate was 20 litres per minute for 15 minutes and resulted in an oxygen concentration of approximately 1 mg per litre, 0.01–0.02% or an oxygen partial pressure of 0.1–0.2 mmHg in the cell culture medium measured with a colorimetric test (VISOCOLOR Eco, Macherey-Nagel, Düren, Germany). After flushing the chamber it was sealed and placed in a regular cell culture incubator for the given time points (24, 48 or 72 hours). To avoid acidification of cell culture media under hypoxic conditions due to CO_2_ withdrawal in the sealed hypoxia chamber, we used CO_2_ independent cell culture media (Invitrogen, Darmstadt, Germany) in all experiments (as well under hypoxic as under control conditions).

### Cloning and plasmid construction

Codon-optimized genes encoding EcFbFP and PpFbFP respectively, were obtained with an upstream Kozak sequence from GeneArt AG (Regensburg, Germany). Genes were synthesized with flanking restriction sites for *Bam*HI and *Eco*RI, which were used to clone the corresponding reporter genes into pCDNA3.1 (Invitrogen, Carlsbad, USA) under the control of the CMV promoter, yielding pCDNA3.1-Bs2 and pCDNA3.1-Pp1, respectively. Afterwards, the genes encoding the FbFP proteins were excised from pCDNA3.1-BS2 and pCDNA3.1-PP1 by usage of *Bam*HI and *Not*I and subsequently cloned into pEF6/Myc-His-lacZ under control of the EF1α promoter, yielding pEF6-Bs2 and pEF6-Pp1. For each step, successful cloning was verified by plasmid preparation, restriction analysis and subsequent sequence analyses. EGFP control vector (pEGFP-C1) is commercially available at BD-Biosciences (BD-Biosciences, Heidelberg, Germany). FMN-based fluorescent proteins (FbFPs) are commercially available under the trademark evoglow at evocatal GmbH (www.evocatal.com).

### Immunfluorescence and microscopy

Cells were seeded on glass cover slips. After the experimental procedure, cells were fixed with Roti Histofix 4% (Carl-Roth, Karlsruhe, Germany) and blocked with 1-fold Roti Immuno Block/0.01% Triton X-100 (Sigma-Aldrich, Munich, Germany). For visualisation of neurons, an antibody against βIII-tubulin (Tuj1; 1∶500, R&D Systems, Wiesbaden-Nordenstadt, Germany) was added and incubated over night at 4°C in 1-fold Roti Immuno Block. For detection of the primary antibody, an indocarbocyanine coupled secondary antibody (Cy3, 1∶800, Chemicon, Billerica, USA) was used in 1-fold Roti Immuno Block.

For visualization of transgene expression, cells were fixed with Roti Histofix 4% (Carl-Roth, Karlsruhe, Germany). All slides were mounted with fluorescent mounting media (Dako, Glostrup, Germany). EGFP expression was visualized with the following filter set: excitation wavelength BP 460–490 nm, emission wavelength BA 510 IF, dichroic mirror 505 nm (Olympus, Hamburg, Germany). For better visualization of FbFPs, a fluorescence filter set with excitation wavelength 450–500 nm, emission wavelength 510–560 nm and a long pass dichroic mirror 470 nm wavelength was used (Chroma, Bellows Falls, USA).

### Determination of average grey values in stably transfected cell lines

Average grey values were used as indicators for fluorescence intensities in stable cell lines. Therefore, all experiments with stably transfected cell lines were performed under the same conditions (seeding density of cells, passage time and media formulation). After the indicated time points of incubation under hypoxic or normoxic conditions, cells were fixated in Roti Histofix 4% (Carl-Roth, Karlsruhe, Germany) and microscope images at the same magnification and exposure time were taken with a black and white camera. Then every cell in the resulting visual field was marked by a ROI (region of interest) and the average grey values were determined by CellR software (Olypmus, Hamburg, Germany). For this calculation each pixel is assigned to a grey value. The lowest grey value (zero) is assigned to black whereas the highest value is assigned to pure white. Afterwards grey values of all pixels in the ROI are summed up and the sum is divided by the amount of pixels in the ROI. The resulting value is the average grey value. Each visual field contained in between 30–100 cells. Grey values of 5–10 visual fields were averaged for calculation of the final average grey values that are displayed in Figure 2 a–c.

### MTT-Assay

To analyze the population extent of msNSC and CHO cells, the optical density, indicative of conversion of 3-(4, 5-dimethylthiazol-2-yl)-2, 5-diphenyltetrazolium bromide (Sigma-Aldrich, Munich, Germany) into formazan crystals which takes place in live cells only, was determined after the indicated time points. Therefore, cells were incubated with MTT (final concentration 0.5 mg/ml) for 3 hours. Afterwards cells were lysed in DMSO (Sigma-Aldrich, Munich, Germany) and the optical density was measured at 560 nm in a spectrophotometer. Cell culture plates used for neural stem cell culture were precoated with poly-L-ornithine (PLO; 0.001%, Sigma-Aldrich, Munich, Germany) and fibronectin (5 µg/ml; Tebu-bio, Offenbach, Germany).
